# Super Dominant Pathobiontic Bacteria in the Nasopharyngeal Microbiota Cause Secondary Bacterial Infection in COVID-19 Patients

**DOI:** 10.1128/spectrum.01956-21

**Published:** 2022-05-17

**Authors:** Tian Qin, Yajie Wang, Jianping Deng, BaoHong Xu, Xiong Zhu, Jitao Wang, Haijian Zhou, Na Zhao, Fangfang Jin, Hongyu Ren, Huizhu Wang, Qun Li, Xinmin Xu, Yumei Guo, Ruihong Li, Yanwen Xiong, XiaoXia Wang, Jiane Guo, Han Zheng, Xuexin Hou, Kanglin Wan, Jianzhong Zhang, Jinxing Lu, Biao Kan, Jianguo Xu

**Affiliations:** a State Key Laboratory for Infectious Disease Prevention and Control, Collaborative Innovation Center for Diagnosis and Treatment of Infectious Diseases, National Institute for Communicable Disease Control and Prevention, Chinese Centers for Disease Control and Prevention, Beijing, China; b Beijing Ditan Hospital, Capital Medical University, Beijing, China; c Centers for Disease Control and Prevention of Zigong City, Zigong, China; d Centers for Disease Control and Prevention of Shijiazhuang City, Shijiazhuang, China; e Central and Clinical Laboratory of Sanya People’s Hospital, Sanya, China; f Centers for Disease Control and Prevention of Taiyuan City, Taiyuan, China; Memorial Sloan Kettering Cancer Center

**Keywords:** pathobiontic bacteria, nasopharyngeal microbiota, secondary bacterial infection, COVID-19

## Abstract

Coronavirus disease 2019 (COVID-19) is a respiratory infectious disease responsible for many infections worldwide. Differences in respiratory microbiota may correlate with disease severity. Samples were collected from 20 severe and 51 mild COVID-19 patients. High-throughput sequencing of the 16S rRNA gene was used to analyze the bacterial community composition of the upper and lower respiratory tracts. The indices of diversity were analyzed. When one genus accounted for >50% of reads from a sample, it was defined as a super dominant pathobiontic bacterial genus (SDPG). In the upper respiratory tract, uniformity indices were significantly higher in the mild group than in the severe group (*P* < 0.001). In the lower respiratory tract, uniformity indices, richness indices, and the abundance-based coverage estimator were significantly higher in the mild group than in the severe group (*P* < 0.001). In patients with severe COVID-19, SDPGs were detected in 40.7% of upper and 63.2% of lower respiratory tract samples. In patients with mild COVID-19, only 10.8% of upper and 8.5% of lower respiratory tract samples yielded SDPGs. SDPGs were present in both upper and lower tracts in seven patients (35.0%), among which six (30.0%) patients possessed the same SDPG in the upper and lower tracts. However, no patients with mild infections had an SDPG in both tracts. Staphylococcus, Corynebacterium, and Acinetobacter were the main SDPGs. The number of SDPGs identified differed significantly between patients with mild and severe COVID-19 (*P* < 0.001). SDPGs in nasopharyngeal microbiota cause secondary bacterial infection in COVID-19 patients and aggravate pneumonia.

**IMPORTANCE** The nasopharyngeal microbiota is composed of a variety of not only the true commensal bacterial species but also the two-face pathobionts, which are one a harmless commensal bacterial species and the other a highly invasive and deadly pathogen. In a previous study, we found that the diversity of nasopharyngeal microbiota was lost in severe influenza patients. We named the genus that accounted for over 50% of microbiota abundance as super dominant pathobiontic genus, which could invade to cause severe pneumonia, leading to high fatality. Similar phenomena were found here for SARS-CoV-2 infection. The diversity of nasopharyngeal microbiota was lost in severe COVID-19 infection patients. SDPGs in nasopharyngeal microbiota were frequently detected in severe COVID-19 patients. Therefore, the SDPGs in nasopharynx microbiota might invade into low respiratory and be responsible for secondary bacterial pneumonia in patients with SARS-CoV-2 infection.

## INTRODUCTION

Severe acute respiratory syndrome coronavirus 2 (SARS-CoV-2), a novel human pathogen, caused the coronavirus disease 2019 (COVID-19) global pandemic that began at the end of 2019 (1). The spread of this virus caused devastating illness and mortality in a short period following hundreds of millions of cases worldwide, resulting in a significant threat to public health and the global economy (2).

Respiratory microbiota is considered guardians of respiratory health and can prevent colonization by invading respiratory pathogens (3). The microbiota of the human nasopharynx is composed of symbiotic and pathogenic bacteria. Depending on environmental conditions, these bacteria may be harmless colonizing microorganisms or highly invasive pathogenic microorganisms (4), including Streptococcus pneumoniae (5), Haemophilus influenzae (6), Neisseria meningitidis (7), and Staphylococcus aureus (8).

In our previous study, we discovered significant differences in the nasopharyngeal microbiota between severe and mild influenza patients, with a super dominant pathobiontic bacterial genus (SDPG) in the nasopharynx of severe influenza patients (9). SDPGs are defined as genera accounting for >50% of sequences in a nasopharyngeal swab, and they are correlated with secondary bacterial infection and death in influenza patients (9). Here, we investigated whether SDPGs in the nasopharyngeal microbiota are associated with a secondary bacterial infection in COVID-19 patients.

## RESULTS

### Infection markers of severe and mild patients.

The procalcitonin (PCT) and C-reactive protein (CRP) values of all mild patients included in this study were normal. For severe patients, PCT and CRP values were higher than normal at sampling time points.

A subsequent bacterial infection was diagnosed in patients displaying elevated levels of PCT or CRP or an isolated pathogenic bacterial species in blood, alveolar lavage fluid, air intubation, or sputum samples. Such infections were recorded for 71% of severe cases and 10% of mild cases (*P* < 0.001).

### Diversity of the respiratory tract bacterial community in COVID-19 patients.

From 178 samples, 11,426,355 high-quality bacterial 16S rRNA sequence reads (Table S2) were obtained with a length range of 407 to 430 bp (mean 422.4 ± 4.5 bp) and 40,417 to 69,999 (mean 64,193) sequences/swab. After clustering sequences into operational taxonomic units (OTUs) and removing OTUs with RA <0.1%, the average number of OTUs per sample was 299.5 ± 159.4. The average numbers of OTUs in the mild upper, mild lower, and severe lower respiratory tract samples were 252.84 ± 36.08, 267.87 ± 36.13, and 386.09 ± 257.36, respectively.

**FIG 1:**
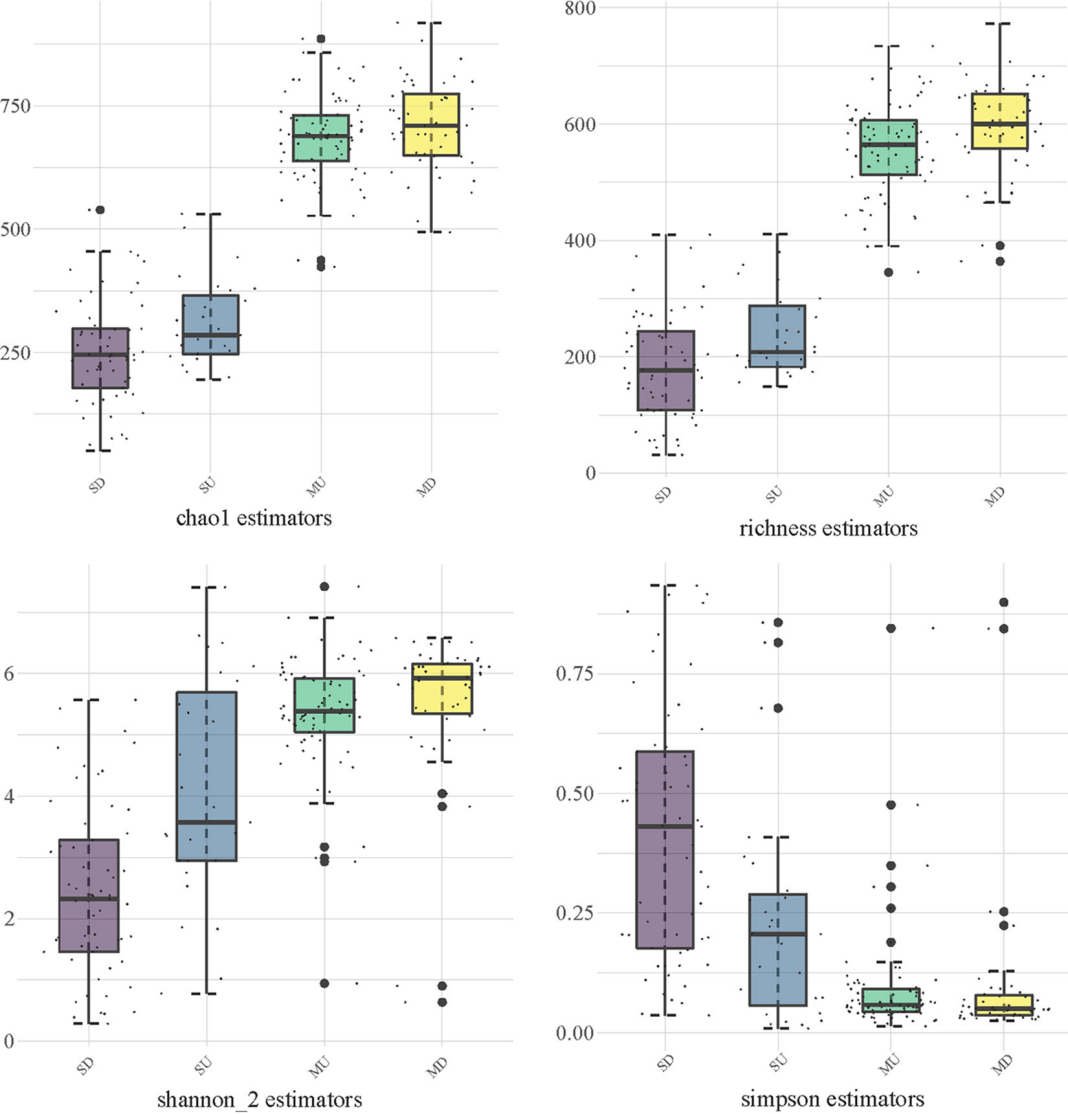
Alpha diversity analysis of bacterial communities in the upper and lower respiratory tracts of severe and mild COVID-19 patients. MU, upper respiratory tract of mild COVID-19 patients; MD, lower respiratory tract of mild COVID-19 patients; SU, upper respiratory tract of severe COVID-19 patients; SD, lower respiratory tract of severe COVID-19 patients.

Six alpha diversity indices were used to analyze the diversity of the bacterial community in the respiratory tracts of the patients. In the upper respiratory tract, three uniformity indices (Shannon, Simpson, and Pielou) were significantly higher in the mild group than in the severe group (*P* < 0.001; Fig. 1). This indicated that, in the upper respiratory tract, the uniformity of the bacterial community was higher in mild COVID-19 cases than in severe cases. In the lower respiratory tract, three uniformity indices (Shannon, Simpson, and Pielou), richness indices (the number of species observed, Chao1, and Richness), and the abundance-based coverage estimator (ACE1) were significantly higher in the mild group than in the severe group (*P* < 0.001; Fig. 1). This indicated that, in the lower respiratory tract, the uniformity and richness of the bacterial community were higher in mild COVID-19 patients than in severe COVID-19 patients. According to the Bray-Curtis distance, the bacterial community compositions of the mild and severe patients were different (Fig. S1).

### Dominant pathogens in the respiratory bacterial microbiota.

Overall, 25 phyla (8 to 17 for each sample), 39 classes (15 to 28 for each sample), 91 orders (27 to 56 for each sample), 159 families (36 to 99 for each sample), and 412 genera (58 to 178 for each sample) were annotated. The top three most abundant phyla were Proteobacteria, Firmicutes, and Bacteroidetes, together accounting for 81.4%, 76.8%, 91.2%, and 72.5% of the sequences in the lower respiratory tract of the mild group, the upper respiratory tract of the mild group, the lower respiratory tract of the severe group, and the upper respiratory tract of the severe group, respectively.

**FIG 2:**
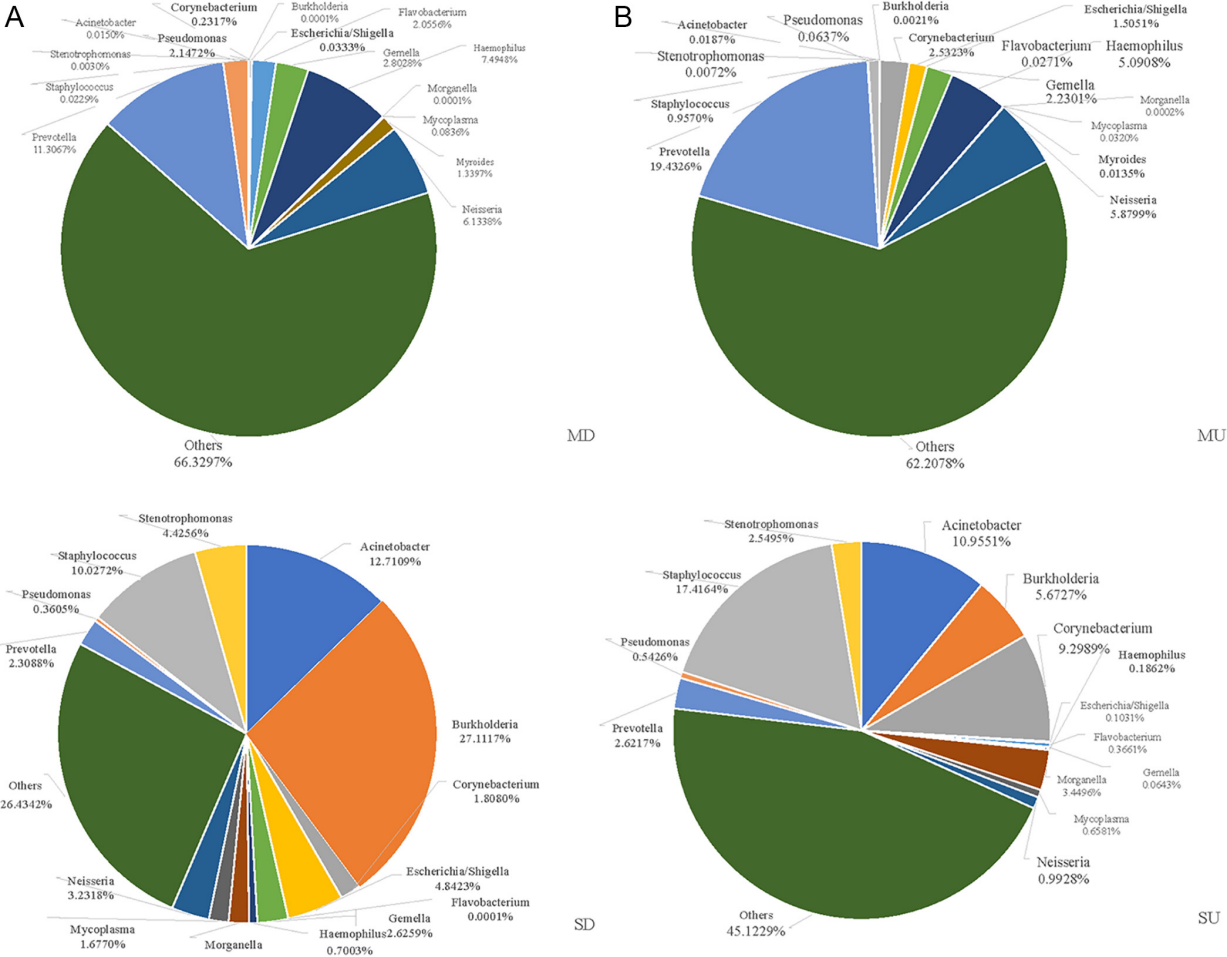
Top 15 most abundant families (A) and genera (B) in the nasopharyngeal microbiota of lower and upper respiratory tracts of mild and severe COVID-19 patients. MU, upper respiratory tract of mild COVID-19 patients; MD, lower respiratory tract of mild COVID-19 patients; SU, upper respiratory tract of severe COVID-19 patients; SD, lower respiratory tract of severe COVID-19 patients.

According to the statistics for the top 15 abundant families, there were great differences between severe and mild patients, while differences between upper and lower respiratory tracts in the same symptom group were minimal (Fig. 2S).

We next compared the relative abundance of the top 15 most dominant genera across the four groups. Similarly, the results showed that differences were great between severe and mild COVID-19 groups and small between upper and lower respiratory tracts within the same symptom groups (Fig. 2). Overall, the relative abundance of Burkholderia, Acinetobacter, Staphylococcus, Escherichia*/*Shigella, Morganella, and Corynebacterium in the severe COVID-19 group was significantly higher than in the mild group (*P* < 0.001).

### SDPGs in the respiratory tract of COVID-19 patients.

**FIG 3:**
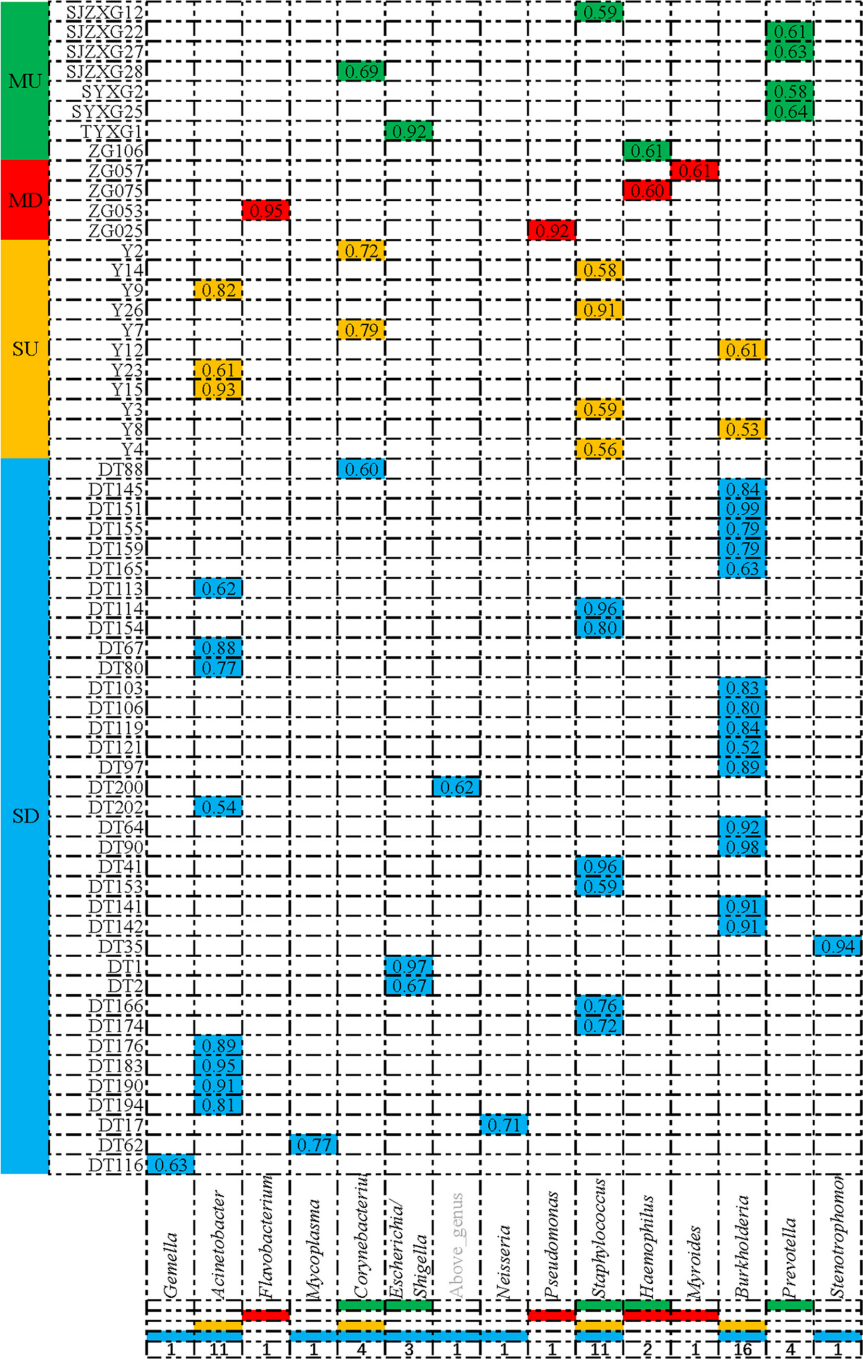
Super dominant pathobiontic bacterial genus (SDPG) identification by sequencing upper and lower respiratory tract samples of mild and severe COVID-19 patients. MU, upper respiratory tract of mild COVID-19 patients; MD, lower respiratory tract of mild COVID-19 patients; SU, upper respiratory tract of severe COVID-19 patients; SD, lower respiratory tract of severe COVID-19 patients.

If a genus was responsible for >50% of reads, it was defined as an SDPG. We found that 10.8% of upper respiratory tract samples from mild patients harbored an SDPG, including Prevotella (*n* = 4), Staphylococcus (*n* = 1), unidentified Corynebacterium (*n* = 1), Escherichia*/*Shigella (*n* = 1) and Haemophilus (*n* = 1). Meanwhile, 40.7% of upper respiratory tract samples from severe patients carried an SDPG, including Staphylococcus (*n* = 4), Acinetobacter (*n* = 3), Corynebacterium (*n* = 2), and Burkholderia (*n* = 2). In total, 8.5% of mild patients included an SDPG in the lower respiratory tract, including Pseudomonas (*n* = 1), Flavobacterium (*n* = 1), Haemophilus (*n* = 1), and Myroides (*n* = 1). Meanwhile, 63.2% of lower respiratory tract samples of severe patients displayed an SDPG, including Burkholderia (*n* = 14), Acinetobacter (*n* = 8), Staphylococcus (*n* = 6), Escherichia*/Shigella* (*n* = 2), Stenotrophomonas (*n* = 1), Mycoplasma (*n* = 1), Neisseria (*n* = 1), Gemella (*n* = 1) and Corynebacterium (*n* = 1; Fig. 3 and Table S3). The proportion of severe patients with SDPGs was significantly higher than that of mild patients (*P* < 0.05), and the proportion of lower respiratory tracts of severe patients was significantly higher than that of upper respiratory tracts (*P* < 0.05). However, the proportion of lower respiratory tracts in mild patients was slightly lower than that of upper respiratory tracts (*P = *0.833).

### Isolation of pathogens from severe lower respiratory tract samples displaying SDPGs.

Pathogens were isolated from 30 severe lower respiratory tract samples containing an SDPG (Table S4). The pathogens were Burkholderia cepacia (*n* = 13), Acinetobacter baumannii (*n* = 5), Staphylococcus aureus (*n* = 4), Stenotrophomonas maltophilia (*n* = 3), Escherichia coli (*n* = 2), Enterobacter cloacae (*n* = 2), and Staphylococcus hominis (*n* = 1). Sequencing results were compared with culture results, and 86.7% (26/30) of bacterial pathogens isolated from severe lower respiratory tract samples were consistent with the sequencing results.

### The same SDPGs are present in the upper and lower respiratory tracts of some severe patients.

**FIG 4:**
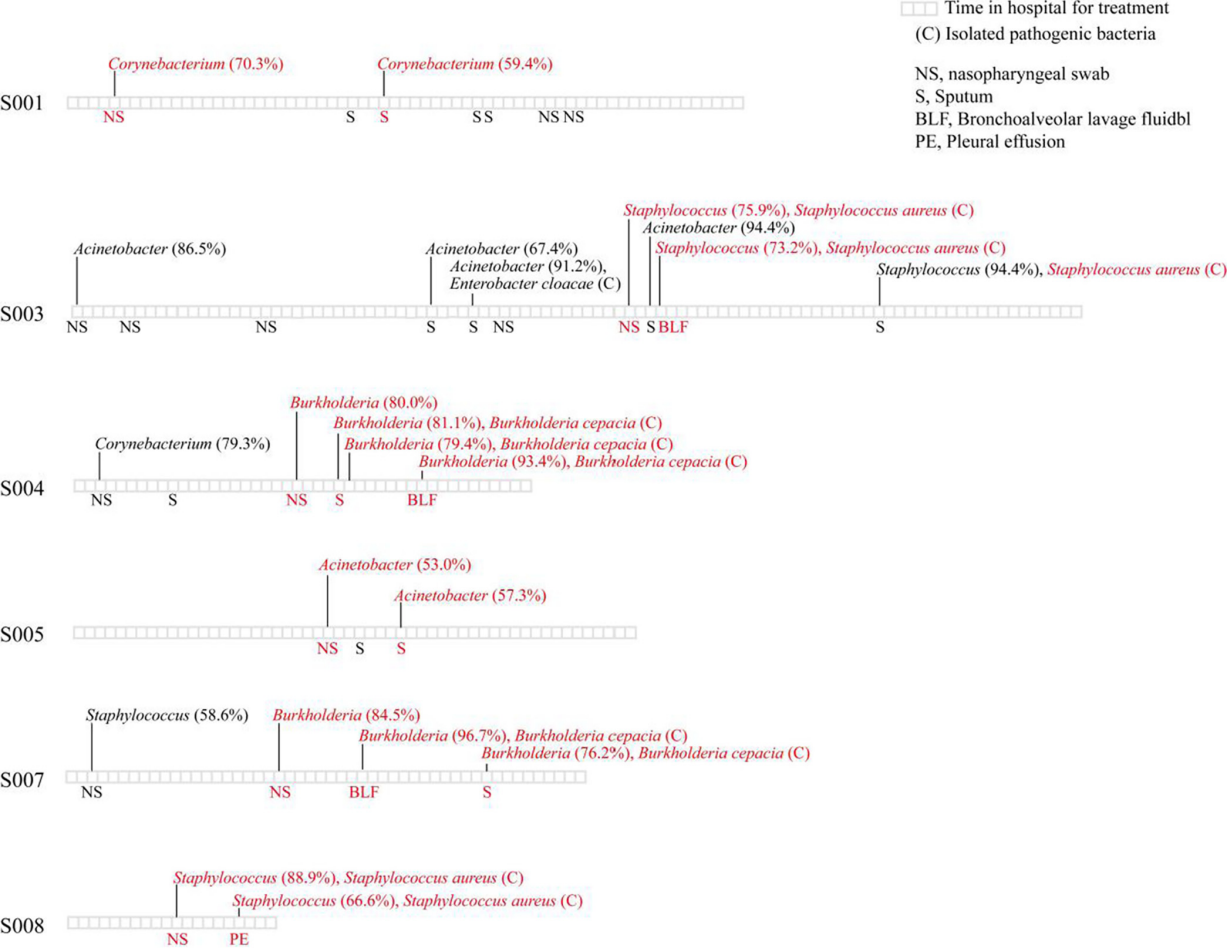
Timeline of cases possessing the same SDPG in the upper and lower respiratory tract.

**FIG 5:**
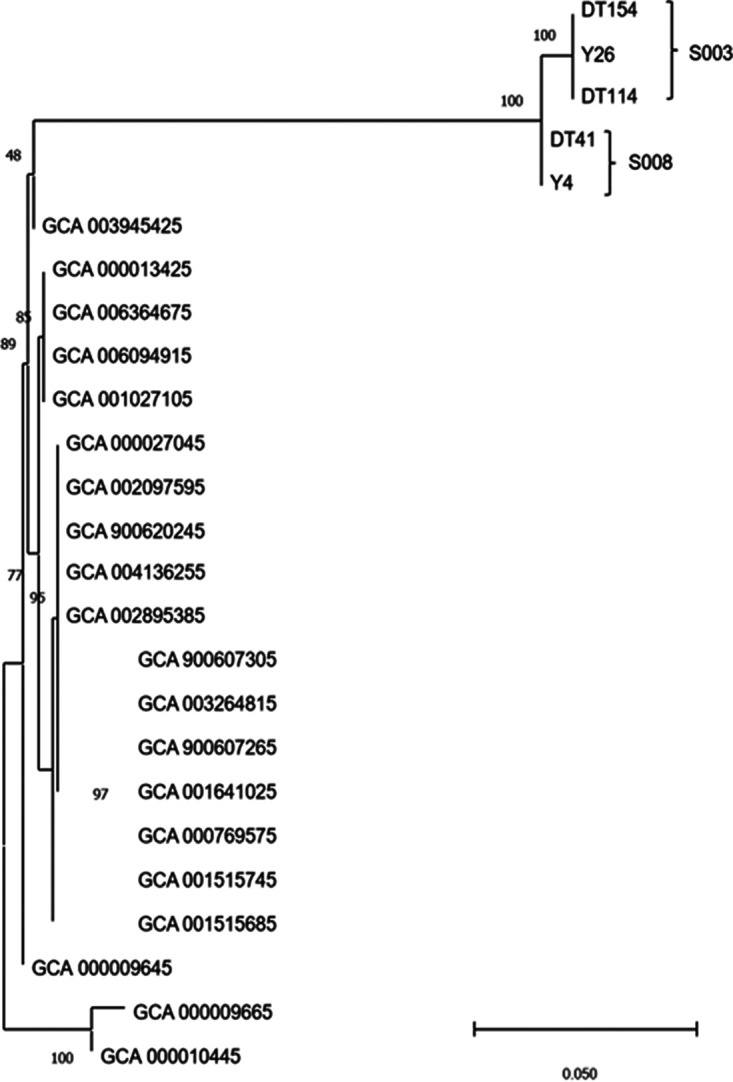
Genome sequences of identical strains isolated from both nasopharyngeal swabs and lower-respiratory tract infection sites.

It was hypothesized that SDPGs emerged due to lateral transfer from the upper respiratory tract to the lower respiratory tract. If this hypothesis is true, SDPGs should be consistent in the upper and lower respiratory tracts of the same patient. In this study, SDPGs were found in both the upper and lower respiratory tracts of seven severe COVID-19 patients. Among them, six patients (cases numbers S001, S003, S004, S005, S007, and S008) had the same SDPG in the upper and lower respiratory tract (Table 1 and Fig. 4). These SDPGs included *Burkholderia* (numbers S004 and S007), Staphylococcus (numbers S003 and S008), Acinetobacter (number S005), and *Corynebacterium* (number S001). Furthermore, in two cases (numbers S003 and S008), S. aureus cells with identical genome sequences were isolated from both the upper and lower respiratory tracts (Fig. 5).

**TABLE 1:** Super dominant pathobiontic bacterial genus (SDPG) identification via sequencing and culturing of pathogens in severe COVID-19 patients

Patient ID	Sample no.	Test date	Type of specimen	SDPG by sequencing	Pathogens obtained by culture
S001	Y2	2020/2/11	Nasopharyngeal swab	*Corynebacterium* (70.3%)	not obtained
	DT78	2020/3/6	Sputum		not obtained
	DT88	2020/3/9	Sputum	*Corynebacterium* (59.4%)	not obtained
	DT107	2020/3/18	Sputum		not obtained
	DT110	2020/3/19	Sputum		not obtained
	Y6	2020/3/25	Nasopharyngeal swab		not obtained
	Y25	2020/3/27	Nasopharyngeal swab		not obtained
S002	DT145	2020/4/9	Sputum	*Burkholderia*	Burkholderia cepacia
	Y14	2020/4/3	Nasopharyngeal swab	Staphylococcus	not obtained
	Y18	2020/4/11	Nasopharyngeal swab		not obtained
	DT151	2020/4/14	Bronchoalveolar lavage fluid	*Burkholderia*	Burkholderia cepacia
	DT155	2020/4/14	Sputum	*Burkholderia*	Burkholderia cepacia
	DT159	2020/4/16	Sputum	*Burkholderia*	Burkholderia cepacia
	DT165	2020/4/20	Sputum	*Burkholderia*	Stenotrophomonas maltophilia
	DT172	2020/4/21	Bronchoalveolar lavage fluid		not obtained
	DT175	2020/4/22	Bronchoalveolar lavage fluid		not obtained
S003	Y9	2020/1/26	Nasopharyngeal swab	Acinetobacter (86.5%)	not obtained
	Y10	2020/1/31	Nasopharyngeal swab		not obtained
	Y11	2020/2/13	Nasopharyngeal swab		not obtained
	DT67	2020/3/2	Sputum	Acinetobacter (67.4%)	not obtained
	DT80	2020/3/6	Sputum	Acinetobacter (91.2%)	Enterobacter cloacae
	Y24	2020/3/9	Nasopharyngeal swab		not obtained
	Y26	2020/3/21	Nasopharyngeal swab	Staphylococcus (75.9%)	Staphylococcus aureus
	DT113	2020/3/23	Sputum	Acinetobacter (94.4%)	not obtained
	DT114	2020/3/24	Bronchoalveolar lavage fluid	Staphylococcus (73.2%)	Staphylococcus aureus
	DT154	2020/4/14	Sputum	Staphylococcus (94.4%)	Staphylococcus aureus
S004	Y7	2020/2/21	Nasopharyngeal swab	*Corynebacterium* (79.3%)	not obtained
	DT65	2020/2/28	Sputum		not obtained
	Y12	2020/3/12	Nasopharyngeal swab	*Burkholderia* (80.0%)	not obtained
	DT97	2020/3/16	Sputum	*Burkholderia* (81.1%)	Burkholderia cepacia
	DT103	2020/3/17	Sputum	*Burkholderia* (79.4%)	Burkholderia cepacia
	DT119	2020/3/24	Bronchoalveolar lavage fluid	*Burkholderia* (93.4%)	Burkholderia cepacia
S005	Y23	2020/5/1	Nasopharyngeal swab	Acinetobacter (53.0%)	not obtained
	DT192	2020/5/4	Sputum		not obtained
	DT202	2020/5/8	Sputum	Acinetobacter (57.3%)	Stenotrophomonas maltophilia
S006	Y15	2020/5/31	Nasopharyngeal swab	Acinetobacter	not obtained
S007	Y3	2020/2/3	Nasopharyngeal swab	Staphylococcus (58.6%)	not obtained
	Y8	2020/2/21	Nasopharyngeal swab	*Burkholderia* (84.5%)	not obtained
	DT64	2020/2/28	Bronchoalveolar lavage fluid	*Burkholderia* (96.7%)	Burkholderia cepacia
	DT90	2020/3/12	Sputum	*Burkholderia* (76.2%)	Burkholderia cepacia
S008	Y4	2020/2/14	Nasopharyngeal swab	Staphylococcus (88.9%)	Staphylococcus aureus
	DT41	2020/2/20	Pleural effusion	Staphylococcus (66.6%)	Staphylococcus aureus
S009	Y16	2020/4/5	Nasopharyngeal swab		not obtained
	DT153	2020/4/14	Sputum	Staphylococcus	Staphylococcus aureus
	DT158	2020/4/16	Sputum		not obtained
	Y21	2020/4/19	Nasopharyngeal swab		not obtained
	DT203	2020/5/11	Sputum		not obtained
S010	DT137	2020/4/1/	Sputum		not obtained
	Y27	2020/4/1	Nasopharyngeal swab		not obtained
	DT140	2020/4/2	Bronchoalveolar lavage fluid		not obtained
	DT141	2020/4/7	Sputum	*Burkholderia*	Burkholderia cepacia
	DT142	2020/4/7	Sputum	*Burkholderia*	Burkholderia cepacia
S011	DT35	2020/2/17	Sputum	*Stenotrophomon*	Stenotrophomonas maltophilia
	Y1	2020/2/20	Nasopharyngeal swab		not obtained
	DT66	2020/3/2	Sputum		not obtained
S012	Y5	2020/2/1	Nasopharyngeal swab		not obtained
	DT1	2020/2/3	Sputum	Escherichia */Shigella*	Escherichia coli
	DT2	2020/2/5	Sputum	Escherichia */Shigella*	Escherichia coli
S013	Y13	2020/4/7	Nasopharyngeal swab		not obtained
	DT144	2020/4/9	Sputum		not obtained
	DT160	2020/4/16	Sputum		not obtained
	DT167	2020/4/20	Sputum		Acinetobacter baumannii
	DT177	2020/4/23	Sputum		not obtained
S014	Y19	2020/4/17	Nasopharyngeal swab		not obtained
	DT166	2020/4/20	Sputum	Staphylococcus	Staphylococcus aureus
	Y17	2020/4/20	Nasopharyngeal swab		not obtained
	DT174	2020/4/21	Bronchoalveolar lavage fluid	Staphylococcus	Staphylococcus aureus
	Y22	2020/4/31	Nasopharyngeal swab		not obtained
S015	DT176	2020/4/22	Sputum	Acinetobacter	Acinetobacter baumannii
	DT183	2020/4/25	Sputum	Acinetobacter	Acinetobacter baumannii
	DT190	2020/4/29	Sputum	Acinetobacter	Burkholderia cepacia
	Y20	2020/4/31	Nasopharyngeal swab		not obtained
	DT194	2020/5/5	Sputum	Acinetobacter	Acinetobacter baumannii
S016	DT17	2020/2/12	Sputum	*Neisseria*	not obtained
S017	DT14	2020/2/11	Sputum		not obtained
S018	DT62	2020/2/27	Sputum	*Mycoplasma*	not obtained
S019	DT115	2020/3/24	Sputum		not obtained
	DT116	2020/3/24	Sputum	*Gemella*	not obtained
	DT117	2020/3/24	Sputum		not obtained
S020	DT134	2020/3/31	Sputum		not obtained

## DISCUSSION

Viral pneumonia is a potentially fatal human disease with severe social and economic impacts, and its prevention and control are a challenge to infectious disease medicine and biosecurity (10, 11). Coronaviruses have caused many outbreaks of viral pneumonia in the last 20 years, including severe acute respiratory syndrome (SARS) in 2003, Middle East respiratory syndrome (MERS) in 2012, and COVID-19 in 2019. Since the outbreak of COVID-19, the severity of pneumonia has greatly increased the medical burden on treatment systems and has even caused their collapse in some areas. Therefore, preventing the development of severe COVID-19 is a key aim. Secondary bacterial infection is an important factor that leads to severe viral pneumonia and directly affects the fatality rate (12, 13). Secondary bacterial infections may occur during patient hospitalization or even in the community before hospitalization.

Several approaches are critical for improving the treatment of patients with COVID-19 pneumonia. First, diagnostic research should be carried out on pathogens responsible for secondary infections in severely ill patients. Second, the characteristics and effects of mixed infections during treatment should be analyzed, and the diagnosis of mixed infections should be established. Third, drug-resistant bacteria should be detected (14). Finally, early warning technology for nosocomial transmission should be developed (15). However, to date, there have been few reports on the source of secondary bacterial infection in viral pneumonia.

The human upper respiratory tract microbiota is generally considered to differ depending on various factors. In healthy individuals, the nasopharyngeal microbiota prevents the colonization of invading respiratory pathogens and is considered a gatekeeper of respiratory health (3). However, in patients with viral respiratory tract infections, bacteria in the nasopharynx may invade the lower respiratory tract, leading to more severe respiratory diseases, and even bacteremia, because the viral infection can alter the nasopharyngeal microbiota through a variety of mechanisms (16). The results of the present study indicated that the bacterial diversity of the upper and lower respiratory tracts was significantly lower in patients with mild COVID-19 than in patients with severe COVID-19. The occurrence of SDPGs was significantly higher in severe patients (40.7% had an SDPG in the upper respiratory tract and 63.2% had an SDPG in the lower respiratory tract) than in mild patients (10.8% had an SDPG in the upper respiratory tract and 8.5% had an SDPG in the lower respiratory tract; *P* < 0.05). Furthermore, there were seven severe COVID-19 patients with the same SDPG in the upper and lower respiratory tract. From these results, it could be proposed that the severity of COVID-19 in patients may be correlated with SDPGs in the respiratory flora. Additionally, SDPG persistence may aggravate the condition of COVID-19 patients. A similar conclusion was drawn for influenza patients (9).

B. cepacia, S. aureus, and A. baumannii were among the main SDPG strains isolated from the lower respiratory tract of severe COVID-19 patients. These bacteria are common conditional pathogens in the respiratory tract that cause severe respiratory infections, especially in intensive care unit (ICU) patients due to critical illness, low immune function, and other risk factors This result not only indicates that SDPG persistence may play a role in secondary bacterial infection after COVID-19 disease but also provides significant guidance for treating severe COVID-19 patients.

There are some limitations to this study. First, the nasopharyngeal microbiota of COVID-19 patients before hospitalization was not clear. Thus, it was not possible to investigate whether these SDPGs were caused by the environment or by increases in the abundance of existing colonization strains. Second, due to the lack of samples from healthy people as control, it is impossible to compare the compositions of the microbiome in upper respiratory tracts between COVID-19 patients and healthy persons. However, there have already been several reports on COVID-19 patients with upper respiratory tract microbiome disorders (17, 18). Third, the use of antibiotics in the treatment of severe patients was unknown, and it was not possible to investigate the reasons for the changes in SDPGs in the lower respiratory tract microbiota over time. Fourth, fungi and viruses could not be determined due to the limits of the methodology used in this work (16S RNA gene sequencing). Fifth, patients from different geographic regions may experience different levels of air pollution, which potentially contributes to microbiome differences in the upper respiratory tract (19, 20). Nevertheless, the results indicate a potential relationship between COVID-19 severity and SDPGs in the respiratory tract.

In conclusion, most severe patients were found to possess an SDPG in their lower respiratory tract, and some were consistent with an SDPG in the upper respiratory tract, according to high-throughput sequencing of the 16S rRNA gene, bacterial culture, and genome analyses. Therefore, we propose that viral infection leads to dysbacteriosis of the upper respiratory tract, causing some pathogens to become SDPGs and then invade the lower respiratory tract, leading to secondary bacterial pneumonia. This is similar to what can occur in influenza patients (9). Therefore, it may be a common phenomenon that SDPGs in the nasopharynx lead to secondary bacterial pneumonia in patients with viral respiratory tract infections.

## MATERIALS AND METHODS

### Experimental design and sample collection.

The studied cases were divided into mild and severe subgroups. All COVID-19 cases were classified as mild based on the COVID-19 Pneumonia Diagnosis and Treatment Plan issued by the National Health Commission of the people’s Republic of China, unless they possessed one or more of the following clinical manifestations, in which case they were classified as severe: (i) shortness of breath, respiratory rate (RR) ≥30 times/min; (ii) in the resting state when inhaling air, oxygen saturation is ≤93%; (iii) arterial blood oxygen partial pressure (PaO_2_)/inhaled oxygen concentration (FiO_2_) ≤300 mm Hg (1 mm Hg = 0.133 kPa); at high altitude (>1000 m), PaO_2_/FiO_2_ should be corrected according to the formula PaO_2_/FiO_2_ × (760/atmospheric pressure [mm Hg]); (iv) clinical symptoms progressively worsening, and lung imaging shows lesions progressing significantly (>50%) within 24 to 48 h.

A total of 51 mild and 20 severe COVID-19 cases were included in this study. Twenty severe COVID-19 cases were hospitalized in Beijing Ditan Hospital, China, from February to May 2020. All severe cases were judged as a secondary bacterial infection because their serum procalcitonin (PCT) and C-reactive protein (CRP) levels were abnormal and indicated bacterial infections. Fifteen severe patients provided throat swabs at least once during hospitalization, and each patient provided sputum and/or bronchoalveolar lavage fluid at least once during hospitalization. For pharyngeal swab samples, we selected the samples at the time of admission. For clinical samples, we collected samples when abnormal PCT and CRP values indicated bacterial infection. Under a 10× microscope, there are less than 10 squamous epithelial cells and more than 25 leukocytes in each field (or the squamous epithelial cells/leukocytes ratio is less than 1:2.5), which was regarded as qualified sputum samples. The 51 mild COVID-19 cases were from Shijiazhuang (25 patients), Sanya (11 patients), Taiyuan (6 patients), and Zigong (9 patients) between January and April 2020. Each mild case provided at least one nasopharyngeal swab during this period and every patient from Zigong provided sputum at least once.

A total of 205 respiratory tract samples were obtained for analysis in this study, including 27 upper and 57 lower respiratory tract samples collected from severe cases (Table 1) and 74 upper and 47 lower respiratory tract samples collected from mild cases (Table S1). Samples were collected from the nasopharynx of each subject using sterile swabs and placed in a collection tube containing 2 mL phosphate-buffered saline (PBS). Sputum or bronchoalveolar lavage fluid was collected in sterile centrifuge tubes, immediately refrigerated, and transported to the laboratory for storage at −80°C.

### Ethics statement.

The studies involving human participants were reviewed and approved by the Ethics Committee of Beijing Ditan Hospital affiliated with Capital Medical University, China, No. (023) – 01. The patients/participants provided written informed consent to participate in this study.

### High-throughput sequencing, annotation, and analysis of 16S rRNA genes.

The cetyltrimethylammonium bromide (CTAB) method was used to extract genomic DNA from samples. The V3−V4 region of the 16S rRNA gene was amplified by PCR for 30 cycles using primers F (5′-CCTAYGGGRBGCASCAG-3′) and R (5′-GGACTACNNGGGTATCTAAT-3′) with high-efficiency high-fidelity polymerase enzyme. A TruSeq DNA PCR-Free Sample Preparation kit was used to construct the library. The library was quantified using Qubit and Q-PCR, and NovaSeq6000 was used for sequencing. According to the barcode sequence and PCR primer sequence, data for each sample were separated from offline data. After removal of barcodes and primer sequences, reads were spliced using FLASH (v1.2.7; http://ccb.jhu.edu/software/FLASH/) to obtain Raw Tags. According to the QIIME tag quality control process (v1.9.1; http://qiime.org/scripts/split_libraries_fastq.html), Raw Tags were strictly filtered to obtain high-quality tags (Clean Tags). The final effective data were obtained by removing chimera sequences (Effective Tags).

Uparse software (Uparse v7.0.1001; http://www.drive5.com/uparse/) was used to cluster all effective tags of all samples. Sequences were clustered into operational taxonomic units (OTUs) using a 97% identity threshold. According to the algorithm principle, the sequence with the highest frequency was selected as the representative sequence for each OTU. OTU sequences were annotated and analyzed using Mothur and the small subunit (SSU) rRNA database of SILVA132 (http://www.arb-silva.de/) with a threshold of 0.8 to 1. Taxonomic information was obtained, and the community composition of each sample was determined at each taxonomic level (kingdom, phylum, class, order, family, genus, and species). The phylogenetic relationships of all OTUs were determined using MUSCLE software (version 3.8.31; http://www.drive5.com/muscle/) to perform rapid multiple sequence alignment. Finally, data for each sample were normalized, and the sample with the least amount of data was used as the standard. Subsequent alpha and beta diversity analyses were based on the normalized data.

QIIME software (version 1.9.1) was used to calculate uniformity indices (Shannon, Simpson, and Pielou), richness indices (the number of species observed, Chao1, and Richness), and the abundance-based coverage estimator (ACE1). R software (version 2.15.3) was used to draw dilution, rank abundance, and species accumulation curves, and to analyze differences in alpha diversity between groups.

To eliminate possible contamination, quality control methods were implemented. OTUs with relative abundance (RA) <0.1% were filtered out. Furthermore, 16 negative controls were tested, including 10 swabs, 3 ddH_2_O, and 3 PBS samples. These blank control samples were used for bacterial genomic DNA extraction and sequencing using the same laboratory, equipment, and batch of reagents and consumables. Possible contamination based on the top 10 OTUs of negative controls was eradicated from the specimen sequence library.

### Isolation and identification of pathogenic bacterial species.

Pathogenic bacterial species from lower respiratory tract samples (alveolar lavage fluid, sputum) and blood of severe patients were isolated and identified (21) using whole-genome sequencing and sequence comparisons (Text S1).

### Statistical analyses.

The rates of subsequent bacterial infection between severe and mild cases were compared using the χ^2^ test with a row × column table (McNemar’s test). Differences in the number of bacteria were analyzed by a nonparametric test of independent samples using SPSS 11.5 software. Differences were considered significant at *P* < 0.05.

### Data availability.

Data are available in a public, open access repository. All sequence data from this study has been submitted to Sequence Read Archive (https://www.ncbi.nlm.nih.gov/sra) and can be accessed through the BioProject ID PRJNA830536.
