# Supplementary material for: Super Dominant Pathobiontic Bacteria in the Nasopharyngeal Microbiota Cause Secondary Bacterial Infection in COVID-19 Patients
Source: Microbiol Spectr. 2022 May 17;10(3):e01956-21. doi: 10.1128/spectrum.01956-21 (PMC9241909; doi:10.1128/spectrum.01956-21)
Supplement: SUPPLEMENTAL FILE 1 — Supplemental material. Download spectrum.01956-21-s001.pdf, PDF file, 1 MB [file spectrum.01956-21-s001.pdf]

Supplemental Methods

Whole-genome sequencing and genetic relatedness analysis of pathobiontic bacteria

Bacterial strains were subjected to Illumina sequencing by constructing two paired-end (PE) libraries with average insertion lengths of 500 bp and 2000 bp. Sequences were generated using an Illumina GA IIx platform (Illumina Inc., San Diego, CA, USA). Raw data were processed in four steps: removing reads with 5 bp of ambiguous bases, removing reads with 20 bp of low quality (≤Q20) bases, removing adapter contamination, and removing duplicated reads. Finally, 100× libraries were obtained with clean PE read data. Single-nucleotide polymorphisms (SNPs) were examined through pairwise comparisons using SOAPsnp[1]. Reads of low quality (>3 consecutive bases with a quality score ≤Q20) were removed before SNP calling. SNPs were called if they met the following criteria using SOAPsnp45: (i) each SNP site was covered by ≥20 reads, (ii) the distance between two SNP sites was ≥5 bp, (iii) the SNP was not located in a repeat region, and (iv) the prior probability of heterozygous SNPs was ≤0.1%.

References for supplemental methods

1. Li R, Li Y, Fang X, et al. SNP detection for massively parallel whole-genome resequencing. *Genome Res*. 2009;19(6):1124-1132.

Supplemental Tables

Table S1. Clinical information for mild COVID-19 patients

Patient ID	Sample No.	Test date	Type of specimen	Sequence number	Test location
MOO1	MUOO1-1	\	Throat swab	SJZ-XG-1	Shijiazhuang
MOO2	MUOO2-1	\	Throat swab	SJZ-XG-2	Shijiazhuang
MOO3	MUOO3-1	\	Throat swab	SJZ-XG-3	Shijiazhuang
MOO4	MUOO4-1	\	Throat swab	SJZ-XG-4	Shijiazhuang
MOO5	MUOO5-1	\	Throat swab	SJZ-XG-5	Shijiazhuang
MOO6	MUOO6-1	\	Throat swab	SJZ-XG-6	Shijiazhuang
MOO7	MUOO7-1	\	Throat swab	SJZ-XG-7	Shijiazhuang
MOO8	MUOO8-1	\	Throat swab	SJZ-XG-8	Shijiazhuang
MOO9	MUOO9-1	\	Throat swab	SJZ-XG-9	Shijiazhuang
MO10	MUO10-1	\	Throat swab	SJZ-XG-10	Shijiazhuang
MO11	MUO11-1	\	Throat swab	SJZ-XG-11	Shijiazhuang
MO12	MUO12-1	\	Throat swab	SJZ-XG-12	Shijiazhuang
MO13	MUO13-1	\	Throat swab	SJZ-XG-13	Shijiazhuang
MO15	MUO15-1	\	Throat swab	SJZ-XG-15	Shijiazhuang
MO16	MUO16-1	\	Throat swab	SJZ-XG-16	Shijiazhuang
MO17	MUO17-1	\	Throat swab	SJZ-XG-17	Shijiazhuang
MO18	MUO18-1	\	Throat swab	SJZ-XG-18	Shijiazhuang
MO20	MUO20-1	\	Throat swab	SJZ-XG-20	Shijiazhuang

MO21	MUO21-1	\	Throat swab	SJZ-XG-21	Shijiazhuang
MO22	MUO22-1	\	Throat swab	SJZ-XG-22	Shijiazhuang
MO23	MUO23-1	\	Throat swab	SJZ-XG-23	Shijiazhuang
MO24	MUO24-1	\	Throat swab	SJZ-XG-24	Shijiazhuang
MO25	MUO25-1	\	Throat swab	SJZ-XG-25	Shijiazhuang
MO27	MUO27-1	\	Throat swab	SJZ-XG-27	Shijiazhuang
MO28	MUO28-1	\	Throat swab	SJZ-XG-28	Shijiazhuang
M030	MU030-1	\	Throat swab	SYXG2	Sanya
	MU031-1	\	Throat swab	SYXG3	Sanya
M031	MU031-2	\	Throat swab	SYXG5	Sanya
M033	MU033-3	\	Throat swab	SYXG16	Sanya
	MU034-2	\	Throat swab	SYXG20	Sanya
M034	MU034-3	\	Throat swab	SYXG25	Sanya
M035	MU035-1	\	Throat swab	SYXG9	Sanya
M036	MU036-2	\	Throat swab	SYXG15	Sanya
M037	MU037-1	\	Throat swab	SYXG17	Sanya
M038	MU038-1	\	Throat swab	SYXG18	Sanya
M039	MU039-1	\	Throat swab	SYXG19	Sanya
M040	MU040-1	\	Throat swab	SYXG24	Sanya
M041	MU041-1	\	Throat swab	SYXG26	Sanya
M042	MUO42-1	2020.1.21	Throat swab	TYXG1	Taiyuan

MO43	MUO43-1	2020.1.24	Throat swab	TYXG2	Taiyuan
MO44	MUO44-1	2020.1.26	Throat swab	TYXG3	Taiyuan
MO46	MUO46-1	2020.1.31	Throat swab	TYXG5	Taiyuan
MO50	MUO50-1	2020.2.3	Throat swab	TYXG9	Taiyuan
MO51	MUO51-1	2020.2.7	Throat swab	TYXG10	Taiyuan
M057	MU057-1	\	Nasopharyngeal swab	ZG007	Zigong
	MU057-4	\	Nasopharyngeal swab	ZG019	Zigong
	MD057-10	\	Sputum	ZG103	Zigong
	MD057-2	\	Sputum	ZG008	Zigong
	MD057-6	\	Sputum	ZG043	Zigong
	MD057-8	\	Sputum	ZG087	Zigong
	MU057-7	\	Throat swab	ZG086	Zigong
	MU057-9	\	Throat swab	ZG102	Zigong
M058	MU058-10	\	Nasopharyngeal swab	ZG056	Zigong
	MU058-12	\	Nasopharyngeal swab	ZG058	Zigong
	MU058-2	\	Nasopharyngeal swab	ZG011	Zigong
	MU058-7	\	Nasopharyngeal swab	ZG041	Zigong
	MU058-8	\	Nasopharyngeal swab	ZG052	Zigong
	MD058-1	\	Sputum	ZG002	Zigong
	MD058-11	\	Sputum	ZG057	Zigong
	MD058-13	\	Sputum	ZG059	Zigong

	MD058-15	\	Sputum	ZG065	Zigong
	MD058-17	\	Sputum	ZG073	Zigong
	MD058-19	\	Sputum	ZG075	Zigong
	MD058-21	\	Sputum	ZG079	Zigong
	MD058-23	\	Sputum	ZG083	Zigong
	MD058-25	\	Sputum	ZG091	Zigong
	MD058-27	\	Sputum	ZG093	Zigong
	MD058-3	\	Sputum	ZG012	Zigong
	MD058-30	\	Sputum	ZG105	Zigong
	MD058-32	\	Sputum	ZG101	Zigong
	MD058-33	\	Sputum	ZG107	Zigong
	MD058-5	\	Sputum	ZG027	Zigong
	MD058-6	\	Sputum	ZG040	Zigong
	MD058-9	\	Sputum	ZG053	Zigong
	MU058-16	\	Throat swab	ZG072	Zigong
	MU058-22	\	Throat swab	ZG082	Zigong
	MU058-26	\	Throat swab	ZG092	Zigong
	MU058-28	\	Throat swab	ZG100	Zigong
	MU058-29	\	Throat swab	ZG104	Zigong
	MU058-31	\	Throat swab	ZG106	Zigong
M059	MD059-1	\	Sputum	ZG001	Zigong

	MD059-7	\	Sputum	ZG089	Zigong
M060	MU060-4	\	Nasopharyngeal swab	ZG044	Zigong
	MD060-1	\	Sputum	ZG004	Zigong
	MD060-11	\	Sputum	ZG097	Zigong
	MD060-5	\	Sputum	ZG045	Zigong
	MD060-7	\	Sputum	ZG071	Zigong
	MD060-3	\	Sputum	ZG029	Zigong
	MD060-9	\	Sputum	ZG085	Zigong
	MU060-10	\	Throat swab	ZG096	Zigong
	MU060-6	\	Throat swab	ZG070	Zigong
	MU060-8	\	Throat swab	ZG084	Zigong
M061	MU061-4	\	Nasopharyngeal swab	ZG048	Zigong
	MU061-8	\	Nasopharyngeal swab	ZG081	Zigong
	MD061-1	\	Sputum	ZG009	Zigong
	MD061-5	\	Sputum	ZG049	Zigong
	MD061-7	\	Sputum	ZG069	Zigong
	MU061-6	\	Throat swab	ZG068	Zigong
M062	MD062-1	\	Sputum	ZG003	Zigong
	MD062-2	\	Sputum	ZG020	Zigong
	MD062-5	\	Sputum	ZG039	Zigong
	MD062-7	\	Sputum	ZG063	Zigong

	MD062-9	\	Sputum	ZG099	Zigong
	MU062-8	\	Throat swab	ZG098	Zigong
M063	MU063-2	\	Nasopharyngeal swab	ZG032	Zigong
	MD063-1	\	Sputum	ZG010	Zigong
	MD063-3	\	Sputum	ZG033	Zigong
	MD063-5	\	Sputum	ZG047	Zigong
	MD063-7	\	Sputum	ZG067	Zigong
	MD063-9	\	Sputum	ZG095	Zigong
	MU063-8	\	Throat swab	ZG094	Zigong
M064	MU064-2	\	Nasopharyngeal swab	ZG016	Zigong
	MD064-1	\	Sputum	ZG005	Zigong
	MD064-3	\	Sputum	ZG025	Zigong
M065	MU065-2	\	Nasopharyngeal swab	ZG013	Zigong
	MU065-3	\	Nasopharyngeal swab	ZG022	Zigong
	MU065-5	\	Nasopharyngeal swab	ZG060	Zigong
	MU065-7	\	Nasopharyngeal swab	ZG076	Zigong
	MD065-1	\	Sputum	ZG006	Zigong
	MD065-6	\	Sputum	ZG061	Zigong
	MD065-8	\	Sputum	ZG077	Zigong

Note: M, mild; MU, mild upper respiratory tract; MD, mild lower respiratory tract.

Table S2. Summary of analysed sequence information

Sample	Q20	GC%	Raw reads	Clean reads	Effective reads %	Number of bases (nt)	Total tags	Average length (nt)	Taxon Tags	Unclassified Tags	Unique Tags	Number of OTUs
DT1	97.92	55	84,367	62,902	74.56	26,973,957	62902	429	62585	1	316	36
DT103	98.06	53.74	98,402	65,037	66.09	27,797,572	65037	427	64056	135	846	327
DT104	97.94	54.09	86,696	69,487	80.15	29,721,590	69487	430	68689	133	665	264
DT106	98.05	54.29	76,711	63,168	82.35	27,038,555	63168	428	62494	21	653	195
DT107	97.96	54.02	95,020	67,544	71.08	28,859,324	67544	428	66516	126	902	563
DT110	97.83	53.82	67,291	59,641	88.63	25,531,100	59641	428	59154	8	479	237
DT113	98	51.14	98,121	65,795	67.05	28,201,909	65795	428	65155	10	630	359
DT114	97.49	50.8	89,129	62,357	69.96	26,714,143	62357	429	61636	9	712	306
DT115	97.33	51.52	90,625	67,251	74.21	28,708,672	67251	429	66144	2	1105	131
DT116	97.45	51.58	82,050	69,226	84.37	29,635,875	69226	429	68048	27	1151	206
DT117	97.8	52.01	76,164	57,069	74.93	24,172,466	57069	429	55501	59	1509	444
DT119	98.04	54.06	83,916	66,317	79.03	28,357,220	66317	427	65452	147	718	439
DT121	97.91	52.7	67,112	48,977	72.98	20,845,783	48977	429	47733	143	1101	763
DT134	97.53	52.38	96,355	60,721	63.02	25,886,284	60721	428	57659	64	2998	219
DT137	97.35	51.14	63,278	40,417	63.87	17,026,377	40417	425	39721	22	674	419

DT14	97.73	50.99	85,547	66,730	78	28,422,000	66730	424	65568	0	1162	132
DT140	97.71	52.23	98,082	60,868	62.06	25,505,323	60868	428	59781	48	1039	426
DT141	97.75	54.07	91,184	67,431	73.95	28,841,628	67431	429	66911	24	496	558
DT142	97.66	53.08	90,999	69,166	76.01	29,409,758	69166	429	68373	74	719	557
DT144	97.96	51.26	82,336	68,515	83.21	29,141,348	68515	422	68015	1	499	203
DT145	96.91	53	113,402	69,115	60.95	28,488,817	69115	428	65363	1647	2105	966
DT151	97.93	54.49	93,751	65,540	69.91	28,075,145	65540	429	65064	92	384	191
DT153	97.88	50.93	81,935	61,877	75.52	26,225,163	61877	429	61079	269	529	490
DT154	97.88	50.06	73,590	58,889	80.02	25,018,164	58889	421	57468	649	772	677
DT155	97.22	51.61	111,169	66,718	60.01	27,805,724	66718	428	62707	2638	1373	859
DT158	97.83	52.73	93,275	69,554	74.57	29,651,432	69554	420	68357	65	1132	304
DT159	97.79	53.58	96,323	61,797	64.16	26,326,495	61797	426	60043	840	914	569
DT160	97.72	52.15	83,670	62,849	75.12	26,778,054	62849	429	61961	31	857	284
DT165	97.91	54.15	86,488	69,670	80.55	29,825,310	69670	427	69032	76	562	373
DT166	97.63	50.83	99,446	65,521	65.89	27,966,996	65521	423	64646	19	856	544
DT167	97.67	52.02	82,013	67,165	81.9	28,513,100	67165	427	66372	2	791	214
DT17	98.12	51.92	81,716	66,865	81.83	28,550,036	66865	429	66384	14	467	350
DT172	98.25	53.33	81,705	65,171	79.76	26,896,491	65171	428	51014	13526	631	619
DT174	97.95	50.72	84,080	60,226	71.63	25,735,760	60226	428	59411	373	442	409
DT175	98.45	53.12	68,642	54,979	80.1	22,817,118	54979	424	49757	4476	746	928
DT176	97.83	51.15	97,814	68,762	70.3	29,533,328	68762	428	68279	58	425	194

DT177	97.91	51.5	85,343	67,220	78.76	28,321,468	67220	428	66620	25	575	184
DT183	98	51.13	92,244	64,190	69.59	27,573,728	64190	426	63695	12	483	252
DT190	97.9	51.28	86,891	68,928	79.33	29,611,897	68928	428	68495	0	433	186
DT192	97.3	50.88	99,409	60,013	60.37	24,690,868	60013	425	55673	547	3793	1160
DT194	98.05	51.68	90,706	64,986	71.64	27,901,843	64986	412	64584	4	398	188
DT2	98.02	54.82	91,110	64,790	71.11	27,779,691	64790	428	64467	6	317	75
DT200	98.35	53.56	82,520	65,864	79.82	28,046,576	65864	424	64665	227	972	743
DT202	98.15	52.27	94,591	63,131	66.74	27,067,519	63131	417	62562	112	457	348
DT203	98.1	53.53	90,395	63,900	70.69	27,020,179	63900	426	61587	19	2294	206
DT35	98.21	54.36	83,304	67,360	80.86	28,882,970	67360	429	66873	8	479	68
DT41	97.75	51.29	99,219	69,743	70.29	29,903,196	69743	428	68990	4	749	68
DT62	97.71	49.37	85,683	65,266	76.17	27,488,232	65266	430	64151	57	1058	397
DT64	98.01	54.3	96,231	68,638	71.33	29,363,025	68638	428	68093	27	518	334
DT65	98.01	53.79	82,517	63,244	76.64	26,557,801	63244	430	62743	5	496	160
DT66	97.78	52.46	88,158	68,878	78.13	29,316,953	68878	411	68284	1	593	103
DT67	97.99	51.22	87,687	60,901	69.45	26,119,202	60901	428	60270	27	604	234
DT78	97.85	53.55	85,080	67,717	79.59	28,910,739	67717	426	67348	3	366	60
DT80	98.26	51.48	86,876	60,463	69.6	25,632,721	60463	428	58108	132	2223	707
DT88	98.43	53.57	91,845	63,984	69.67	26,658,416	63984	429	63233	55	696	489
DT90	98.14	54.37	85,940	66,930	77.88	28,631,699	66930	429	66424	17	489	377
DT97	97.9	53.11	97,352	60,084	61.72	25,426,096	60084	429	57946	726	1412	913

SJZXG1	97.84	52.89	89,225	64,532	72.33	27,154,243	53278	421	52475	0	803	259
SJZXG10	98.07	51.28	96,972	66,754	68.84	28,387,854	58322	420	57593	0	729	249
SJZXG11	97.79	51.42	81,159	65,200	80.34	27,447,569	54337	418	53704	0	633	272
SJZXG12	97.45	48.7	83,464	52,232	62.58	21,534,753	49239	415	48294	0	945	208
SJZXG13	97.83	51.24	83,925	68,133	81.18	28,282,847	57553	421	56925	0	628	233
SJZXG15	97.53	51.78	84,311	65,610	77.82	27,834,453	46991	421	43746	0	3245	294
SJZXG16	97.84	51.15	93,155	61,814	66.36	26,032,569	41026	420	40596	0	430	247
SJZXG17	97.75	51.54	95,562	64,526	67.52	27,278,155	39457	422	38344	1	1112	285
SJZXG18	97.8	51.17	90,546	65,473	72.31	27,394,444	35252	416	34669	0	583	222
SJZXG2	97.9	51.75	84,977	64,013	75.33	26,861,159	40835	425	40089	0	746	271
SJZXG20	97.86	50.46	67,843	53,515	78.88	22,589,664	38454	421	37533	0	921	292
SJZXG21	97.79	51.34	96,470	66,276	68.7	27,384,733	64164	412	62377	0	1787	236
SJZXG22	97.89	51.44	88,772	67,875	76.46	28,603,977	60166	415	59583	0	583	232
SJZXG23	97.81	51.18	94,621	62,050	65.58	26,133,820	45493	424	44612	0	881	320
SJZXG24	97.82	51.96	86,769	61,875	71.31	26,008,943	48555	421	47495	0	1060	272
SJZXG25	97.74	52.26	97,897	65,063	66.46	27,447,688	43497	423	42942	0	555	225
SJZXG27	97.71	51.28	95,302	68,859	72.25	28,992,113	56493	418	55833	4	656	237
SJZXG28	97.99	52.21	96,205	60,363	62.74	24,541,466	55574	422	54477	0	1097	298
SJZXG3	97.82	51.16	86,313	63,954	74.1	26,729,825	47479	413	45761	0	1718	244
SJZXG4	98.04	50.42	98,666	60,358	61.17	25,030,843	36163	421	35646	0	517	212
SJZXG5	97.81	51.08	92,916	61,333	66.01	25,804,131	38114	421	37310	0	804	243

SJZXG6	97.97	52.14	86,538	60,637	70.07	25,545,997	50904	420	50191	0	713	227
SJZXG7	97.81	51.61	86,847	65,530	75.45	27,501,674	49576	422	48939	0	637	211
SJZXG8	97.94	51.46	97,271	61,238	62.96	25,820,063	51145	421	50613	0	532	261
SJZXG9	97.95	50.14	97,000	61,829	63.74	25,751,351	33040	407	32570	0	470	236
SYXG15	97.77	51.93	98,525	66,823	67.82	28,120,404	44681	422	43507	0	1174	260
SYXG16	97.78	50.79	87,733	66,403	75.69	27,857,287	43670	421	42152	0	1518	267
SYXG17	98.06	50.4	89,152	63,124	70.8	26,491,775	46272	420	45719	0	553	242
SYXG18	97.72	50.84	95,919	67,165	70.02	28,143,532	53084	421	52305	0	779	272
SYXG19	97.86	50.5	92,893	67,650	72.83	28,337,345	31797	421	31229	0	568	233
SYXG2	97.98	51.97	96,088	64,808	67.45	27,376,956	35880	420	35396	0	484	232
SYXG20	97.62	51.91	83,102	60,605	72.93	25,591,594	28316	420	26105	0	2211	256
SYXG24	97.61	52.89	93,116	67,543	72.54	28,648,769	42754	419	40564	0	2190	218
SYXG25	97.89	51.62	81,855	65,930	80.54	27,872,565	15501	419	13576	0	1925	218
SYXG26	97.72	51.22	83,862	63,237	75.41	26,468,092	39656	422	38826	63	767	266
SYXG3	97.76	52.21	89,601	64,656	72.16	27,230,681	40577	424	39935	0	642	224
SYXG5	97.74	51.76	93,611	67,612	72.23	28,392,848	46729	423	45507	0	1222	221
SYXG9	97.73	51.38	82,871	60,918	73.51	25,642,800	37301	419	36150	0	1151	257
TYXG1	97.71	54.76	97,937	69,712	71.18	29,836,822	67486	428	67130	0	356	211
TYXG10	97.86	51.72	96,536	64,854	67.18	27,255,621	40817	423	40187	0	630	225
TYXG2	97.71	51.83	83,662	60,494	72.31	25,611,149	34144	420	33477	0	667	200
TYXG3	98.02	50.97	96,774	63,355	65.47	26,602,428	51983	424	51396	2	585	238

TYXG5	97.87	53.69	92,267	67,569	73.23	28,636,179	54775	422	53205	0	1570	218
TYXG9	97.92	54.11	98,902	67,233	67.98	28,377,278	57783	420	57085	0	698	170
ZG001	97.61	51.34	94,446	62,095	65.75	26,222,704	34746	422	34168	0	578	233
ZG002	97.71	51.6	96,159	61,688	64.15	25,907,007	40974	420	40065	0	909	272
ZG003	97.7	51.88	98,137	69,780	71.1	29,394,629	50997	421	49765	0	1232	235
ZG004	97.93	51.54	99,137	60,623	61.15	25,431,126	38852	419	38297	0	555	254
ZG005	97.78	52.2	95,315	61,723	64.76	25,768,914	43373	417	42778	0	595	250
ZG006	97.76	51.23	96,383	66,204	68.69	27,682,881	52350	418	51775	0	575	272
ZG007	97.86	50.71	97,532	62,400	63.98	25,876,119	38831	415	38383	0	448	219
ZG008	97.83	51.99	91,676	69,943	76.29	29,610,485	43191	423	42428	0	763	246
ZG009	97.72	50.81	84,982	67,274	79.16	28,378,390	38260	422	37631	0	629	280
ZG010	97.79	51.22	93,033	62,848	67.55	26,268,511	34171	418	33677	0	494	198
ZG011	97.84	51.84	96,115	61,128	63.6	25,755,087	43949	421	43502	0	447	232
ZG012	97.91	51.35	93,259	66,852	71.68	27,976,217	51895	418	51182	0	713	277
ZG013	97.86	51.49	87,360	69,659	79.74	29,244,307	48210	420	47714	0	496	193
ZG016	96.53	52.65	214,871	64,405	29.97	26,839,111	58758	422	51953	0	6805	236
ZG019	97.79	52.56	85,814	68,935	80.33	29,070,296	53657	423	53152	0	505	212
ZG020	97.8	52.6	93,887	66,050	70.35	27,928,257	39257	420	38112	0	1145	239
ZG022	97.95	52.31	90,291	62,037	68.71	25,825,587	44661	419	43604	0	1057	281
ZG025	96.41	50.44	90,244	69,999	77.57	29,933,012	67236	422	66871	0	365	189
ZG027	97.85	51.05	92,912	69,371	74.66	28,841,262	51825	419	50641	0	1184	248

ZG029	97.88	52.65	80,831	67,231	83.17	28,204,252	42859	421	41876	0	983	254
ZG032	97.95	50.94	82,085	61,080	74.41	25,594,949	35127	420	34513	0	614	202
ZG033	97.81	51.8	82,510	61,854	74.97	26,107,566	39095	422	38480	0	615	212
ZG039	97.92	52.52	81,950	55,356	67.55	23,200,686	34755	417	31638	0	3117	302
ZG040	97.79	51.11	96,500	62,492	64.76	26,337,030	35107	418	34563	0	544	302
ZG041	97.63	51.8	88,122	60,161	68.27	25,292,430	42410	421	41689	0	721	261
ZG043	97.86	50.82	80,990	67,311	83.11	28,393,890	42227	423	41731	0	496	213
ZG044	97.93	51.18	88,478	68,852	77.82	28,724,197	38839	415	37954	0	885	299
ZG045	97.94	51.47	87,143	66,932	76.81	27,978,619	44117	421	43545	1	571	294
ZG047	97.76	52.22	88,829	64,466	72.57	27,117,527	41064	420	40509	0	555	264
ZG048	97.7	52.26	84,464	67,680	80.13	28,605,263	36685	424	35415	0	1270	282
ZG049	97.88	50.13	86,422	67,403	77.99	28,003,085	58041	418	56697	0	1344	314
ZG052	97.86	51.27	98,053	66,635	67.96	28,003,325	53988	415	53176	0	812	304
ZG053	97.77	46.38	98,039	66,665	68	28,239,806	65483	423	65188	0	295	213
ZG056	97.9	53.07	94,781	60,445	63.77	25,282,963	46723	419	45289	1	1433	314
ZG057	97.81	47.94	87,256	67,762	77.66	28,665,049	58428	423	58039	1	388	315
ZG058	98.04	53.2	84,111	60,732	72.2	25,180,842	51596	417	49740	77	1779	330
ZG059	97.66	51.02	85,931	62,613	72.86	26,474,792	48829	408	48297	1	531	307
ZG060	97.81	50.43	86,063	67,590	78.54	28,349,047	52588	421	52031	1	556	307
ZG061	97.64	51.11	98,664	62,984	63.84	26,660,869	43603	418	42930	1	672	305
ZG063	97.95	50.58	98,153	60,646	61.79	25,301,092	54186	421	52382	0	1804	356

ZG065	97.78	49.87	86,365	46,220	53.52	18,857,829	37320	420	32440	0	4880	289
ZG067	97.83	51.74	94,336	68,675	72.8	28,939,928	37943	421	36869	1	1073	319
ZG068	97.89	51.43	84,676	59,762	70.58	25,004,211	38005	418	36209	4	1792	336
ZG069	97.78	51.72	93,077	58,969	63.36	24,825,121	45775	422	43472	68	2235	327
ZG070	97.79	51.39	96,464	60,495	62.71	25,385,052	38748	427	37099	0	1649	303
ZG071	97.89	51.41	93,489	69,436	74.27	29,260,725	39804	416	39184	1	619	289
ZG072	97.88	51.16	86,235	51,505	59.73	21,547,231	36488	420	34107	0	2381	344
ZG073	97.64	51.63	95,171	65,009	68.31	27,450,768	45471	417	44918	0	553	292
ZG075	97.47	51.5	81,341	61,102	75.12	26,071,131	17030	424	16609	0	421	226
ZG076	97.95	50.33	99,666	60,844	61.05	25,299,884	51262	423	50425	0	837	232
ZG077	97.9	50.75	98,358	69,497	70.66	29,207,543	48949	423	48355	0	594	272
ZG079	97.7	50.31	96,948	61,584	63.52	25,669,555	41258	416	40326	0	932	289
ZG081	97.73	52.37	89,301	68,127	76.29	28,910,162	53913	428	53123	0	790	272
ZG082	97.74	51.59	81,772	67,153	82.12	28,377,429	30937	416	30452	0	485	265
ZG083	97.79	51.63	95,183	62,481	65.64	26,452,122	34477	423	33771	0	706	288
ZG084	97.75	51.96	84,667	60,739	71.74	25,664,896	37525	421	37035	0	490	269
ZG085	97.85	51.67	86,039	61,927	71.98	26,065,680	40103	420	39522	0	581	268
ZG086	97.93	51.02	92,209	64,722	70.19	27,196,995	38705	421	38163	0	542	221
ZG087	97.97	51.53	95,059	68,118	71.66	28,655,360	49345	422	48217	0	1128	261
ZG089	97.98	51.39	93,393	60,644	64.93	25,576,240	44877	425	44298	0	579	243
ZG091	97.73	51.9	83,685	63,847	76.29	27,103,296	31938	423	31437	0	501	244

ZG092	97.86	51.63	83,516	69,836	83.62	29,513,930	44472	422	43992	0	480	241
ZG093	98	51.54	87,060	69,794	80.17	29,480,302	46905	422	46269	0	636	291
ZG094	97.74	51.44	99,284	61,387	61.83	25,920,660	39653	420	38919	0	734	264
ZG095	97.77	51.12	97,095	68,674	70.73	28,847,017	48192	421	47507	0	685	277
ZG096	97.81	51.45	82,095	62,925	76.65	26,461,844	41657	421	41161	0	496	247
ZG097	97.8	51.53	90,317	60,539	67.03	25,497,057	41186	421	40555	0	631	266
ZG098	97.79	51.93	98,521	67,857	68.88	28,575,499	47430	422	46589	0	841	256
ZG099	97.89	51.47	81,808	64,448	78.78	27,184,472	37694	419	36957	0	737	253
ZG100	97.8	51.12	86,132	68,292	79.29	28,637,068	46165	422	45452	0	713	303
ZG101	97.81	51.44	84,413	61,625	73	25,992,493	43267	418	42669	0	598	253
ZG102	97.94	50.66	91,992	66,204	71.97	27,670,406	42058	425	40958	0	1100	238
ZG103	97.75	52.43	81,839	69,589	85.03	29,586,694	56371	423	55732	0	639	213
ZG104	97.81	51.75	87,752	61,254	69.8	25,891,279	37347	423	36768	0	579	294
ZG105	97.74	51.66	91,723	68,733	74.94	29,107,396	40038	425	39502	0	536	291
ZG106	97.57	51.46	89,686	64,068	71.44	27,247,179	18222	422	17853	0	369	239
ZG107	97.88	51.52	83,934	62,090	73.97	26,190,835	44615	417	43902	0	713	295

Note: OTU, operational taxonomic unit.

Table S3. Super-dominant pathobiontic bacterial genera (SDPG) and their relative abundance (RA) in nasopharyngeal microbiota.

Group and case ID	SDPG	RA
Mild upper respiratory tract		
SJZXG12	Staphylococcus	58.5%
SJZXG22	Prevotella	60.7%
SJZXG27	Prevotella	63.5%
SJZXG28	Corynebacterium	69.0%
SYXG2	Prevotella	57.8%
SYXG25	Prevotella	64.3%
TYXG1	Escherichia/Shigella	91.9%
ZG106	Haemophilus	60.6%
Mild lower respiratory tract		
ZG057	Myroides	61.4%
ZG075	Haemophilus	59.6%
ZG053	Flavobacterium	94.8%
ZG025	Pseudomonas	91.9%
Severe upper respiratory tract		
Y15	Acinetobacter	92.6%
Y2	Corynebacterium	70.7%
Y23	Acinetobacter	60.5%

Y26	Staphylococcus	91.3%
Y3	Staphylococcus	59.3%
Y7	Corynebacterium	79.5%
Y9	Acinetobacter	82.4%
Y14	Staphylococcus	57.8%
Y12	Burkholderia	61.4%
Y8	Burkholderia	53.4%
Y4	Staphylococcus	55.8%
Severe lower respiratory tract		
DT97	Burkholderia	88.7%
DT113	Acinetobacter	62.0%
DT114	Staphylococcus	95.7%
DT116	Gemella	62.9%
DT119	Burkholderia	84.0%
DT141	Burkholderia	90.7%
DT142	Burkholderia	90.7%
DT145	Burkholderia	84.0%
DT151	Burkholderia	99.2%
DT153	Staphylococcus	59.0%
DT154	Staphylococcus	79.5%
DT155	Burkholderia	78.9%

DT159	Burkholderia	78.6%
DT165	Burkholderia	63.4%
DT166	Staphylococcus	75.9%
DT174	Staphylococcus	71.6%
DT176	Acinetobacter	89.1%
DT183	Acinetobacter	94.8%
DT190	Acinetobacter	91.1%
DT194	Acinetobacter	80.8%
DT202	Acinetobacter	53.7%
DT41	Staphylococcus	95.7%
DT103	Burkholderia	82.7%
DT106	Burkholderia	79.8%
DT1	Escherichia/Shigella	96.6%
DT2	Escherichia/Shigella	66.9%
DT17	Neisseria	70.7%
DT35	Stenotrophomonas	94.0%
DT62	Mycoplasma	76.5%
DT64	Burkholderia	92.3%
DT67	Acinetobacter	87.9%
DT80	Acinetobacter	77.4%
DT88	Corynebacterium	60.3%

DT90	Burkholderia	98.4%
DT121	Burkholderia	51.9%
DT200	Above_genus	62.1%

Table S4. Pathogens identified and cultured from severe lower respiratory tract SDPG samples

No.	Type of sample	Cultured pathogen	SDPG	Consistency between SDPG and culture results
DT41	Pleural effusion	*Staphylococcus hominis*	*Staphylococcus*	Consistent
DT153	Sputum	*Staphylococcus aureus*	*Staphylococcus*	Consistent
DT145	Sputum	*Burkholderia cepacia*	*Burkholderia*	Consistent
DT151	Alveolar lavage fluid	*Burkholderia cepacia*	*Burkholderia*	Consistent
DT155	Sputum	*Burkholderia cepacia*	*Burkholderia*	Consistent
DT159	Sputum	*Burkholderia cepacia*	*Burkholderia*	Consistent
DT165	Sputum	*Stenotrophomonas maltophilia*	*Burkholderia*	Inconsistent
DT176	Sputum	*Acinetobacter baumannii*	*Acinetobacter*	Consistent
DT183	Sputum	*Acinetobacter baumannii*	*Acinetobacter*	Consistent
DT190	Sputum	*Burkholderia cepacia*	*Acinetobacter*	Inconsistent
DT194	Sputum	*Acinetobacter baumannii*	*Acinetobacter*	Consistent
DT67	Sputum	*Acinetobacter baumannii*	*Acinetobacter*	Consistent
DT80	Sputum	*Enterobacter cloacae*	*Acinetobacter*	Consistent
DT113	Sputum	*Enterobacter cloacae*	*Acinetobacter*	Consistent
DT114	Alveolar	*Staphylococcus aureus*	*Staphylococcus*	Consistent

	lavage fluid			
DT154	Sputum	*Acinetobacter baumannii*	*Staphylococcus*	Inconsistent
DT97	Sputum	*Burkholderia cepacia*	*Burkholderia*	Consistent
DT103	Sputum	*Burkholderia cepacia*	*Burkholderia*	Consistent
DT106	Sputum	*Burkholderia cepacia*	*Burkholderia*	Consistent
DT119	Alveolar lavage fluid	*Burkholderia cepacia*	*Burkholderia*	Consistent
DT202	Sputum	*Stenotrophomonas maltophilia*	*Acinetobacter*	Inconsistent
DT64	Alveolar lavage fluid	*Burkholderia cepacia*	*Burkholderia*	Consistent
DT90	Sputum	*Burkholderia cepacia*	*Burkholderia*	Consistent
DT1	Sputum	*Escherichia coli*	*Escherichia*	Consistent
DT2	Sputum	*Escherichia coli*	*Escherichia*	Consistent
DT141	Sputum	*Burkholderia cepacia*	*Burkholderia*	Consistent
DT142	Sputum	*Burkholderia cepacia*	*Burkholderia*	Consistent
DT166	Sputum	*Staphylococcus aureus*	*Staphylococcus*	Consistent
DT174	Alveolar lavage fluid	*Staphylococcus aureus*	*Staphylococcus*	Consistent
DT35	Sputum	*Stenotrophomonas maltophilia*	*Stenotrophomonas*	Consistent

Supplementary Figure

(A) (B)

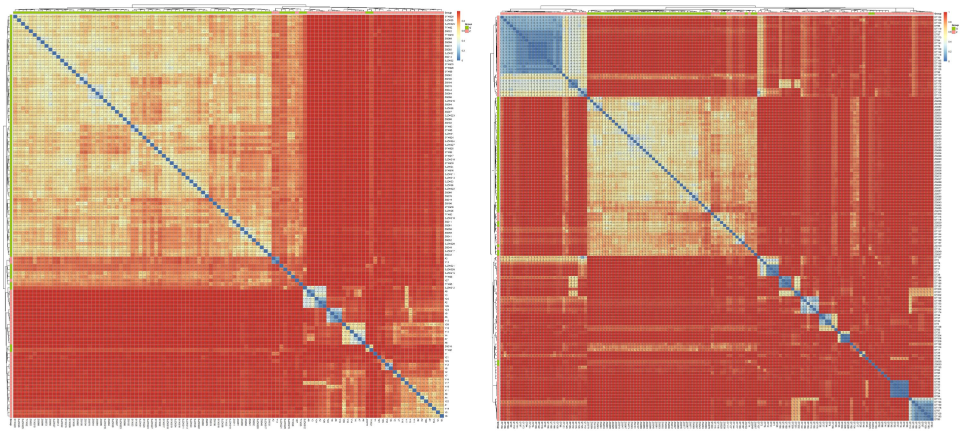

Supplementary Figure 1. Bray-Curtis distances between the bacterial communities of mild and severe XG patients in the upper respiratory tract (A) and lower respiratory tract (B).

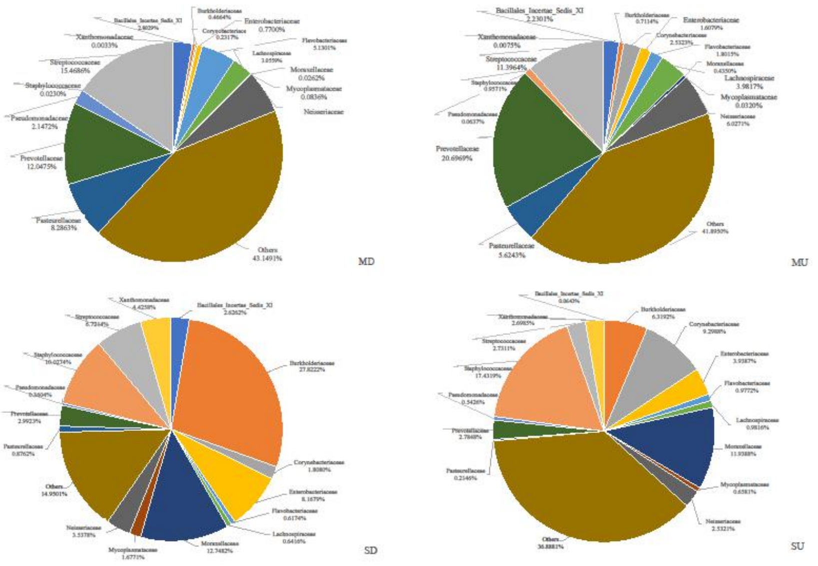

Supplementary Figure 2. Top 15 abundant family in the nasopharyngeal microbiota of lower respiratory tract and upper respiratory tract of mild and severe COVID-19 patients. MU, upper respiratory tract of mild COVID-19 patients; MD, lower respiratory tract of mild COVID-19

patients; SU, upper respiratory tract of severe COVID-19 patients; SD, lower respiratory tract of severe COVID-19 patients.
